# Epigenetic Regulation of ABA-Induced Transcriptional Responses in Maize

**DOI:** 10.1534/g3.119.400993

**Published:** 2020-03-16

**Authors:** Stefania Vendramin, Ji Huang, Peter A. Crisp, Thelma F. Madzima, Karen M. McGinnis

**Affiliations:** * Department of Biological Science, Florida State University, Tallahassee, Florida 32306; † Department of Plant and Microbial Biology, University of Minnesota, St Paul, Minnesota, 55108; ‡ Division of Biological Sciences, School of STEM, University of Washington Bothell, Bothell, Washington, 98011

**Keywords:** Abscisic acid (ABA), *Mediator of paramutation1 (Mop1)*, *Zea mays*, epigenetics, siRNAs

## Abstract

Plants are subjected to extreme environmental conditions and must adapt rapidly. The phytohormone abscisic acid (ABA) accumulates during abiotic stress, signaling transcriptional changes that trigger physiological responses. Epigenetic modifications often facilitate transcription, particularly at genes exhibiting temporal, tissue-specific and environmentally-induced expression. In maize (*Zea mays*), MEDIATOR OF PARAMUTATION 1 (MOP1) is required for progression of an RNA-dependent epigenetic pathway that regulates transcriptional silencing of loci genomewide. MOP1 function has been previously correlated with genomic regions adjoining particular types of transposable elements and genic regions, suggesting that this regulatory pathway functions to maintain distinct transcriptional activities within genomic spaces, and that loss of MOP1 may modify the responsiveness of some loci to other regulatory pathways. As critical regulators of gene expression, MOP1 and ABA pathways each regulate specific genes. To determine whether loss of MOP1 impacts ABA-responsive gene expression in maize, *mop1-1* and *Mop1* homozygous seedlings were subjected to exogenous ABA and RNA-sequencing. A total of 3,242 differentially expressed genes (DEGs) were identified in four pairwise comparisons. Overall, ABA-induced changes in gene expression were enhanced in *mop1-1* homozygous plants. The highest number of DEGs were identified in ABA-induced *mop1-1* mutants, including many transcription factors; this suggests combinatorial regulatory scenarios including direct and indirect transcriptional responses to genetic disruption (*mop1-1*) and/or stimulus-induction of a hierarchical, cascading network of responsive genes. Additionally, a modest increase in CHH methylation at putative MOP1-RdDM loci in response to ABA was observed in some genotypes, suggesting that epigenetic variation might influence environmentally-induced transcriptional responses in maize.

As sessile organisms, plants must adapt rapidly to fluctuating and often extreme abiotic stress conditions that negatively impact crop productivity and yield, such as water deprivation/drought, high salinity, nutrient deficiency and extreme temperatures. In addition to its role in plant development, the phytohormone abscisic acid (ABA) serves as a critical regulator of plant responses to specific abiotic stresses. ABA responses to environmental stress stimuli result in physiological changes primarily directed to minimize water loss, such as stomatal closure and growth inhibition (reviewed by Fujita *et al.* 2011; Sah *et al.* 2016; Kuromori *et al.* 2018).

The signaling events and molecular mechanisms of ABA-mediated responses have been extensively characterized in the model plant *Arabidopsis thaliana* (Arabidopsis). Briefly, under specific abiotic stress, ABA binds to a PYRABACTIN RESISTANCE (PYR)/PYR1-LIKE (PYL)/REGULATORY COMPONENT OF ABA RECEPTOR (RCAR) (PYR/PYL/RCAR) receptor complex which inactivates the negative signaling PHOSPHATASE TYPE 2C (PP2C) protein. Receptor binding by ABA and inactivation of PP2C in turn allows for the positive regulator SNF1-RELATED PROTEIN KINASE 2 (SnRK2) to phosphorylate and activate ABSCISIC ACID RESPONSIVE ELEMENT BINDING PROTEIN/BINDING FACTOR (AREB/ABF) transcription factors (TFs) (reviewed by Mehrotra *et al.* 2014). ABA-induced transcription factors regulate many primary target genes by recognizing and binding to specific ABA-responsive *cis*-elements in their promoters. The promoters of ABA-responsive genes are expected to contain two or more copies of a sequence known as an ABA-Responsive Element (ABRE) (Marcotte *et al.* 1989) or one ABRE that works in conjunction with a Coupling Element (CE) (Shen and Ho 1995; Shen *et al.* 1996) that function as TF binding sites. TFs that contain these regulatory elements can be transcriptionally induced by ABA and trigger the regulation of primary or secondary ABA target genes. Therefore, transcription factor activation, promoter recognition, and DNA-binding activities are critical downstream ABA-mediated physiological responses to abiotic stress.

Transcription factor families that mediate plant responses to abiotic stresses include the APETALA2/ethylene-responsive element-binding proteins (AP2/EREBPs), basic leucine zipper (bZIP), Myeloblastosis (MYB), WRKY and No apical meristem, ATAF1 and CUC cup-shaped cotyledon (NAC) proteins (reviewed by Hoang *et al.* 2017). In several documented examples, transgenic plants overexpressing these regulatory proteins exhibit increased stress tolerance phenotypes (reviewed by Fujita *et al.* 2011; Todaka *et al.* 2015; Joshi *et al.* 2016; Hoang *et al.* 2017), demonstrating the utility of manipulating regulatory factors to increase agricultural productivity under extreme environmental conditions.

Transcription factor binding and transcriptional activation of some abiotic stress-responsive genes relies on alterations to chromatin structure and epigenetic modifications. The relationship between transcription and chromatin remodeling in response to abiotic stress and ABA signaling has recently begun to be appreciated (reviewed by Mehrotra *et al.* 2014; Kim *et al.* 2015; Asensi-Fabado *et al.* 2017; Lämke and Bäurle 2017).

In plants, RNA-dependent DNA methylation (RdDM) is a specific transcriptional gene-silencing pathway that functions to direct changes in epigenetic modifications and gene expression at target loci (reviewed by Matzke *et al.* 2015). The RdDM pathway is reliant on the biogenesis of and response to small non-coding RNAs (sRNAs) through the activity of several proteins, which include two plant-specific DNA-dependent RNA polymerases (Pol IV and Pol V), an RNA-dependent RNA polymerase (RDR), a Dicer-like protein, an Argonaute-complex, DNA modifying enzymes to induce cytosine methylation, ATP-dependent chromatin remodeling complexes, and other accessory proteins (Matzke and Mosher 2014).

In maize, MEDIATOR OF PARAMUTATION 1 (MOP1), an ortholog of the Arabidopsis RDR2 (Dorweiler *et al.* 2000; Alleman *et al.* 2006), interacts with Pol IV (Haag *et al.* 2014) and is required for Pol IV-dependent sRNA biogenesis and progression of the RdDM pathway. A Pol IV-MOP1-dependent pathway maintains chromatin boundaries between protein-coding genes and gene-proximal transposable elements (Li *et al.* 2015a). The *mop1-1* mutation results in a reduction of 24-nt siRNAs and a loss in DNA methylation at certain loci, particularly in the CHH context (Lisch *et al.* 2002; Woodhouse *et al.* 2006; Nobuta *et al.* 2008; Jia *et al.* 2009; Li *et al.* 2014); this mutant stock has proven to be a valuable genetic resource and has been used extensively to characterize Pol IV-mediated RdDM activities across the maize genome (Nobuta *et al.* 2008; Jia *et al.* 2009; Gent *et al.* 2014; Li *et al.* 2014; Madzima *et al.* 2014; Li *et al.* 2015a). Nonetheless, the identification of MOP1 and RdDM targets is not a straight-forward task, because multiple factors contribute to MOP1 target specificity and loss of gene silencing, which initiates a cascade of transcriptional and epigenetic changes that complicate the distinction between MOP1 direct and indirect targets.

Cytosine DNA methylation is important in many biological processes including regulation of transposon silencing and expression of endogenous genes, plant development, and environmental-stress responses (Zhang *et al.* 2018). In plants, this conserved epigenetic mark occurs in symmetric (CG, CHG) and asymmetric contexts (CHH); in which H can be A,T, or C) (Henderson and Jacobsen 2007). CG and CHG methylation is maintained throughout DNA replication by METHYLTRANSFERASE 1 (MET1) and CHROMOMETHYLASE 3 (CMT3) respectively (Niederhuth and Schmitz 2017). Asymmetric methylation can be established by DOMAINS REARRANGED METHYLASE 1/2 (DRM1/2) through the RdDM pathway (Law and Jacobsen 2010).

The majority of the maize genome is composed of transposons (Schnable *et al.* 2009), which are mostly methylated in a symmetrical context (West *et al.* 2014). On a genome-wide scale, methylated CHH sites are observed at significantly lower levels than methylated CG and CHG sites, however, regions of elevated CHH methylation are observed upstream of the transcription start sites of genes (Gent *et al.* 2013). These RdDM-dependent CHH methylation sites coincide with the presence of specific classes of transposons found within 1 kb of many maize genes (Gent *et al.* 2013; Regulski *et al.* 2013; Li *et al.* 2015a).

In Arabidopsis, four glycosylases have been characterized as functioning in active DNA demethylation by initiating removal of methylated cytosines from DNA via base excision repair: REPRESSOR OF SILENCING 1 (ROS1), DEMETER (DME), DME-like 2 (DML2) and DML3 (Choi *et al.* 2002; Agius *et al.* 2006; Gehring *et al.* 2006; Morales-Ruiz *et al.* 2006; Penterman *et al.* 2007). ROS1 has been shown to act through an auto-regulatory, coordinated RdDM-demethylation based response (Lei *et al.* 2015; Williams *et al.* 2015). At some loci, maintenance of transcription appears to be dependent on RdDM activity, which challenges the canonical model of RdDM-mediated transcriptional repression. ROS1 was also recently shown to be required for transcriptional activation of specific ABA-inducible genes in Arabidopsis (Kim *et al.* 2019). The maize DNG103 is highly similar to Arabidopsis ROS1. As reported with *Ros1* in Arabidopsis RdDM mutants (Huettel *et al.* 2006; Li *et al.* 2012), *Dng103* is downregulated in maize RdDM mutants (Jia *et al.* 2009; Madzima *et al.* 2014; Erhard *et al.* 2015; Williams *et al.* 2015). Transcriptional repression of *Dng103* in *mop1-1* mutants likely induces other expression changes that cannot be directly attributed to loss of MOP1 activity.

Plant responses to abiotic stress are multifaceted, integrating multiple environmental cues and biological response mechanisms. These complex mechanisms must be understood to maximize stress tolerance in plants. As both MOP1 and ABA signaling networks act as key regulators of gene expression, characterizing these responses and identifying overlap between the two processes will provide a wealth of information about the mechanisms of stress response in crop plants and candidate pathways to enhance stress response under different agricultural conditions. In this study, we use RNA- and sequence-capture bisulfite sequencing of ABA-induced *mop1-1* mutant and wildtype *Mop1* seedlings to identify MOP1-dependent, ABA-induced genes.

## Materials and Methods

### Plant materials

Maize (*Zea mays*) plants were grown in 2.8 cu. ft potting soil (10-gal coarse sand:400 ml 18.6.12 slow release fertilizer) in greenhouse conditions (16 h light period, 25°, 50% humidity) until they reached the V3 stage (RNA-seq) or ten-day old seedlings (methylation). The *mop1-1* mutation was from genetic stocks from J. Dorweiler of the *mop1-1* mutation introgressed into the B73 reference genome and backcrossed for seven generations (Madzima *et al.* 2014). For this study, progeny resulting from the self-pollination of an ear of a heterozygous plant was used. Only homozygous wildtype and homozygous mutant siblings were used.

### Plant genotyping

The *Mop1/mop1-1* segregating family was genotyped under the following PCR conditions: 94° for 5 min (1x∼; 95° for 30 s, 56° for 45 s, 72° for 45 s (30x); 72° for 10 min (1x); hold for 4° (as needed); using the following primers as previously described (Madzima, Huang, & Mcginnis 2014):

KM384: (5′-TCTCCACCGCCCACTTGAT-3′);

KM385: (5′-CCCAAGAGCT GTCTCGTATCCGT-3′);

KM386: (5′ -CTTCATCTCGAAGTAGCGCTTGTTGTCC-3′).

### Abscisic acid treatment in seedlings

ABA concentrations used in maize seedling studies range from 1 to 150 µM, and 50 µM is commonly used in stage V3 seedlings (∼2 weeks old) (Fan *et al.* 2016; Phillips and Ludidi 2017; Belda-Palazon *et al.* 2018; He *et al.* 2018; Wang *et al.* 2018; He *et al.* 2019). Maize seedlings at the V3 stage were removed from the soil, roots were rinsed in water, dried, and then submerged in a 1 L beaker with 250 mL of liquid Murashige and Skoog (MS) media (Sigma Aldrich, M6899) with 50 μM ABA (ABA; (Sigma Aldrich, (+/−) Abscisic Acid, A1049)) or without ABA (MS) for 7 and 8 hr (methylation and RNA-seq, respectively) in greenhouse conditions (16 h light period, 25°, 50% humidity). After the incubation period, roots where removed and seedlings were immediately flash frozen in liquid nitrogen and stored at -80° until use. Homozygous wildtype (*Mop1* WT) and homozygous *mop1* mutant (*mop1-1*) individuals were used.

### Total RNA isolation

Frozen seedling tissue was finely ground into powder in liquid nitrogen and homogenized before total RNA extraction was performed using TRI reagent according to manufacturer’s instructions (Molecular Research Center, 18080-051). RNA samples were DNase treated (RQ1 RNase-Free DNase, Promega, M6101) and purified using RNA clean & concentrator 25 (Zymo Research, R1018). The integrity and quality of the total RNA was checked by a NanoDrop 1000 spectrophotometer and by 1% agarose gel electrophoresis.

### RNA library preparation, RNA-sequencing and read alignment

Three biological replicates were used for all RNA-seq experiments for each treatment and genotype, for a total of 12 samples. The final sample concentration was quantified by Qubit. RNA library preparation (NEBNext Ultra II kit, NEB, E7760) and Illumina paired-end 150 bp (PE 150) sequencing were performed by Novogene Corporation (Sacramento, California). More than 20 million reads were obtained per library, FASTQ adapters were trimmed by Cutadapt 1.8.1 (Martin 2011) and quality control was performed using FASTQC v0.11.2 (bioinformatics.babraham.ac.uk/projects/fastqc/). Reads were mapped to the B73 maize genome (AGP B73v4) (Jiao *et al.* 2017) by HISAT2 v2.04 (Pertea *et al.* 2016). Transcripts were assembled *de novo* to allow for inclusion of transcripts that are not included in the reference genome annotation and quantified using StringTie v1.3.4d (Pertea *et al.* 2016). Gene count matrices were generated from this data using the prepDE.py python script available in the StringTie website (http://ccb.jhu.edu/software/stringtie/index.shtml?t=manual). These matrices were used by the Bioconductor package edgeR 3.20.9 (Chen *et al.* 2016) in R for differential expression analysis in order to identify upregulated and downregulated genes for the four different genotypes under two treatments. Low-abundance counts of < 0.58 cpm were removed using the DESeq2 filtering method (statquest.org/2017/05/16/statquest-filtering-genes-with-low-read-counts/); (Love *et al.* 2014) incorporated into the edgeR pipeline, and genes with an adjusted p-value of ≤ 0.05 and an absolute log_2_ fold change (FC) value of ≥ 0.95 were considered as differentially expressed for both upregulated and downregulated genes.

### Reverse Transcription Quantitative PCR (RT-qPCR) analysis of transcript abundance

Reverse Transcription Quantitative PCR (RT-qPCR) was performed for all 12 RNA samples to confirm an induction of ABA treatment by measuring the expression of *Responsive to ABA* 17 (*Rab17)* (Vilardell *et al.* 1990) in maize seedlings. The well characterized ABA and stress-responsive maize gene *Viviparous1* (*Vp1*) (Cao *et al.* 2007) was used as a negative control, since it is only ABA-responsive in maize embryos, and *Ubiquitin conjugase* was used as a control. *Rab17* qPCR primers KM1634 (5′-TCCTTGATTCCCTTCTTCCTC-3′) and KM1635 (5′-GAGGGAGGAGCACAAGACC-3′), *Vp1* qPCR primers KM1465 (5′- CCGATGTCAAGTCGGGCAAATATC-3′) and KM1466 (5′-TGGAACCACTGCCTTGCTCTTG-3′), and *Ubiquitin conjugase* KM633 (5′- GACTACACGATGGAGAACATCCTAACCC-3′) and KM634 (5′- GAAGAATGTCCCTTCTGGAGGCTGC-3′) were used.

RT-qPCR was performed for 11 of the 12 RNA samples (excluding a wild type control that had atypical expression patterns and was therefore excluded as an outlier) to measure gene expression of *Dng101* (Zm000010a020199), *Dng103* (Zm00001d038302), and *Dng105* (Zm00001d016521). The following qPCR primers were used for *Dng101*, KM1656 (5′-CTTCTGCTCTTGCTGCTCCA-3′) and KM1657 (5′-CGACTGAAGAGATA CAACGATGC-3′), *Dng103* KM1467 (5′- ACCATGCTGTGACCCTCAAATGG-3′) and KM1468 (5′- CATAGCTGTTCGACAAGGAACCAG-3′), and *Dng105* KM1658 (5′-GGCAAGATATACAGGAATGCTTGA-3′) and KM1659 (5′- CGATGTCCTAGTCCGCTTCA -3′). *Ubiquitin conjugase* was used as a control amplified by primers KM63 5′- GACTACACGATGGAGAACATCCTAACCC-3′) and KM634 (5′- GAAGAATGTCCCTTCTGGAGGCTGC-3′).

First-strand cDNA synthesis and RT-qPCR were performed by Florida State University’s Biology Molecular Core Facility. First-strand cDNA synthesis was performed by reverse transcribing 1 μg of total RNA with Oligo(dT)_20_ primers according to manufacturer’s instructions (SuperScriptTM III Reverse Transcriptase, Invitrogen, 18080-051). Reverse transcriptase quantitative PCR (RT-qPCR) was performed using an Applied Biosystems 7500 Fast Real-Time PCR System and SYBR Green reagents (Thermofisher Scientific). The generation of specific PCR products was confirmed by melting curve analysis. Primers were designed using MaizeGDB (maizegdb.org) and Net Primer Analysis Software (premierbiosoft.com/netprimer/).

### DNA methylation library construction, sequencing, and analysis

High molecular weight genomic DNA was isolated from above ground tissue of maize 10-day old seedlings using a standard CTAB protocol. Sequence capture library construction used 0.5-1 μg of genomic DNA as previously described by (Li *et al.* 2015b). Adapters were trimmed using Trim Galore! (https://www.bioinformatics.babraham.ac.uk/projects/trim_galore/). Reads were aligned to maize genome version 4 (AGPv4) using *BSMAP-2.90* (-v 5 -q 20) (Xi & Li 2009) and uniquely mapped reads were processed as described previously (Han *et al.* 2018) The unmethylated chloroplast genome was used to determine cytosine conversion rate. The methylation ratio (number of reads that were methylated and unmethylated) was determined for each methylation context by the weighted DNA methylation method (#C / (#C + #T)) using *BSMAP* tools. BEDTools and overlapping reads were used to determine read coverage for target regions.

Genome-wide methylation changes were determined by assessing regions with a minimum of 5x coverage and 2 methylation sites, and methylation levels of 10% for CG and CHG contexts and 1% for the CHH context. Methylation % difference was calculated by the equation: ((Genotype/Treatment 1 mC% - Genotype/Treatment 2 mC%) / (Genotype/Treatment 2)).

Promoter regions of DEGs were defined as the 2 kb region upstream from the transcription start site. This 2000 bp region was divided into 100 bp tiles for further methylation analysis. Tiles with no data were removed and not counted for the final average mC level. Coverage was calculated using DNA methylation data in 100 bp tiles. Methylation percentage was defined as [treatment – control] x100. For average methylation % in the promoter region of DEGs, a minimum of 2x coverage, 2 methylation sites, 10% methylation for CG/CHG and 1% for CHH was used.

Pairwise comparisons for *Mop1* wildtype ABA *vs.* *Mop1* wildtype MS (Figure 6, comparison A) and *mop1-1* mutant MS *vs.* *Mop1* wildtype MS (Figure 6, comparison D) were correlated with genes in Group I and II, and Groups III, IV, V and VI from RNA-Seq results, respectively. Differentially methylated promoters were identified by comparisons where there was an absolute methylation difference (Genotype/Treatment 1 mC% - Genotype/Treatment mC 2%) of ≥ 40% in the CG/CHG context; in the CHH context where one Genotype/Treatment sample showed a methylation level of ≤ 5% and between comparisons a methylation % difference higher than ≥15%. A region with ≥10% methylation increase in the CG context was defined as having CG hypermethylation.

### Gene ontology analysis

Gene ontology (GO) (Plant GO Slim) analysis was conducted using the web-based tool agriGO v2.0 (Tian *et al.* 2017), incorporating AGOv4 IDs into a Single Enrichment Analysis (SEA) using the Fisher’s statistical test, Hochberg (FDR) multi-test adjustment method with a significance level of < 0.05 and minimum number of mapping entries of 10 genes per GO-term. The GO term enrichment was generated by hierarchically clustering the log10 of the total GO term percentage of a set of genes that were upregulated or downregulated in wildtype or mutant in response to ABA (Table S4).

### Alignment and overlap of 24 nt siRNA and promoter proximal regions of DEGs

Using publicly available data from NCBI’s Gene Expression Omnibus (GEO, GSE68510; (Wang *et al.* 2017)) from (*Mop1*/*mop1-1*) wildtype and mutant (*mop1-1*/*mop1-1*) maize young cob small RNA raw reads were processed for 24-nt siRNA alignment and comparison. The TruSeq Small RNA 3′ adapter (RA3) sequences were removed from the raw reads using Cutadapt (Martin 2011) and quality of the reads was assessed before and after the trimming using FASTQC v0.11.2 (bioinformatics.babraham.ac.uk/projects/fastqc/). Alignment of the reads was performed by ShortStack v3 (Johnson *et al.* 2016) using default parameters. Using the (.bam) alignment files generated by ShortStack v3 and the chromosomal location of the 2 kb region upstream of the transcription start site (TSS) (downloaded using biomart ensembl plants) (ShortStack specified parameter:–loci) of all protein coding genes in *Zea mays* (B73 RefGen_v4), the promoter proximal regions of these genes were assessed for the presence of 24 nt siRNAs. Differential 24 nt siRNA targeting between *Mop1* wildtype and *mop1-1* mutant in this 2 Kb region upstream of TSS was calculated with edgeR 3.20.9 (Robinson *et al.* 2010; Mccarthy *et al.* 2012) after removal of low-abundance regions and library normalization. Further normalization was performed to the foldchange of 2.2 (log_2_ of 1.13), calculated using the abundances of the undisturbed expression of high-confidence maize miRNAs from miRbase (http://www.mirbase.org/cgi-bin/mirna_summary.pl?org=zma; B73_RefGen_v4) in the mutant and wildtype (miRNA abundances in *mop1-1* mutant/miRNA abundances in *Mop1* wildtype; Table S4, File S6).

### ABA-response transcription factor network

Based on the Arabidopsis ABA-responsive transcription factor hierarchical network built using chromatin immunoprecipitated (ChIP) data (Song *et al.* 2016) maize orthologs for the genes present in the network were identified using a list generated by blasting maize proteins against TAIR 10 protein sequences using standalone BLASTP version 2.2.28+ 581 (Camacho *et al.* 2009) (File S3). Gene targets for transcription factors located in every level or tier were predicted using a tissue-specific gene regulatory network in seed, SAM, root and leaf maize tissues (https://www.bio.fsu.edu/mcginnislab/mgrn/) (Huang *et al.* 2018) and were represented as edges. Nodes represent the log_2_FC of these genes in our dataset (Figure S5).

### Motif discovery and enrichment analysis

The Multiple Expectation Maximization (EM) for Motif Elicitation (MEME) tool (http://meme-suite.org) version 5.0.4 was used to search for the presence of any conserved domains among the ABA-response transcription factor network built from our dataset. The promoter sequences of 47 genes were used with the following parameters: -dna -mod anr -revcomp -minw 5 -maxw 10 -nmotifs 10 -evt 0.05. A motif highly similar to the maize ABRE was found in 66% of these genes. Find Individual Motif Occurrences (FIMO) version 5.0.5 was used to identify ABA-responsive genes in other sets of DEGs. Motif enrichment was performed using the previously-found putative ABRE motif (CACG[TC]G[TG]C[GC]) as input. Primary ABA targets were defined as genes that were differentially expressed in response to ABA and that contained at least two ABRE sites in their promoter or one ABRE site and a coupling element (CE).

### Transposable element enrichment analysis

Bedtools ‘closest’ tool was used to identify enriched TEs adjacent to the 2 kb upstream or 2 kb downstream DNA region of DEGs from groups V and VI (File S10). Gene annotations from the differentially expressed genes of interest were extracted from the B73 Refgen_v4 genome assembly (Jiao *Et Al*., 2017) using EnsemblPlants BioMart (plants.ensembl.org/biomart/) (Kinsella *et al.* 2011). The transposable element (TE) annotation file used was downloaded from github (B73.structuralTEv2.2018.12.20.filteredTE.gff3.gz; https://github.com/SNAnderson/maizeTE_variation). The percentage distribution of transposable element families in the B73 genome was calculated using from data from previously published studies (Anderson *et al.* 2019).

### Data availability

All RNA-sequencing and Bisulfite-Sequencing data are available at the National Center for Biotechnology Information (NCBI) Gene Expression Omnibus (Edgar *et al.* 2002) through GEO Series accession number GSE132167.

Figure S1: Rab17 RT-qPCR

Figure S2: MDS clustering

Figure S3: DNG RT-qPCR

Figure S4: *Mop1* WT and *mop1-1* mutant promoter methylation for DEGs and non-DEGs

Figure S5: Heatmap (Log2 FC) of TF Network DEGs

Table S1: DEGs no. for DE analysis using different biological replicates no.

Table S2: Maize protein phosphatases class A (*PP2C*-A)

Table S3: Number of GO terms found for DEGs in four groups

Table S4: High-confidence miRNAs

File S1: Groups I-VII log2FC

File S2: GO term enrichment per DEG group

File S3: Homologous TFs

File S4: Tiers and Downstream genes

File S5: Group model parameters per gene

File S6: Genome-wide siRNA changes in *mop1-1* mutant

File S7: SeqCap DNA methylation ratios in all sequence contexts

File S8: Promoter DNA methylation for *Mop1* Wildtype ABA-responsive DEGs

File S9: MOP1-ABA targets with a loss of siRNA and DNA methylation at ABRE sites

File S10: TE enrichment in a subset of genes in Groups V and VI.

Supplemental material available at figshare: https://doi.org/10.25387/g3.9626636.

## Results

### Canonical abiotic stress associated genes are transcriptionally responsive to ABA in wildtype and mop1-1 maize seedlings

To determine the combined effects of MOP1 and ABA on gene expression in maize, homozygous wildtype (*Mop1*) and mutant (*mop1-1)* individuals from a segregating family of seedlings at vegetative stage 3 (V3) from a self-pollinated *Mop1/mop1-1* ear were subjected to treatment with ABA. Induction was confirmed by measuring the expression of the *Responsive to ABA 17 (Rab17)* gene (Figure S1), which encodes a putative dehydrin protein and has been the subject of study for ABA- and osmotic stress-responsiveness. Upregulation of *Rab17* expression is well-documented in several transcriptomic studies measuring responses to ABA and drought in maize embryos (Vilardell *et al.* 1990; Goday *et al.* 1994) and vegetative tissues (Vilardell *et al.* 1994; Busk and Pages 1998; Zheng *et al.* 2004) as well as in drought studies in sorghum leaves at the seedling stage (Johnson *et al.* 2014). Reverse transcription and quantitative PCR (RT-qPCR) of *Rab17* confirmed that the ABA-treatment of V3 seedlings was sufficient to induce changes in gene expression (Figure S1).

RNA from these ABA-induced and control genotypes was subjected to cDNA library preparation, RNA-sequencing and transcriptome analysis. An average of ∼23 million 150 bp paired end raw reads were obtained per sample and mapped to the B73 reference genome (AGP B73v4) (Jiao *et al.* 2017). Mapped reads were used in subsequent analysis (Table 1). Multidimensional scaling (MDS) was performed to determine the relationship between genotype and treatment for the 12 homozygous *Mop1* and *mop1-1* samples, with the majority clustering according to genotype and treatment (Figure S2). One biological replicate of a *Mop1* wildtype sample that was not treated with ABA clustered with the wildtype ABA-treated samples. This suggests non-experimental endogenous induction of ABA in this seedling, perhaps related to a stress response induced in this individual under control treatment conditions. Due to evidence of an ABA-induced response, this sample was removed from further analysis and only two replicates for the wildtype control were used. The removal of wt_m1_ms_3 did not affect the proportion of differentially expressed genes (DEGs) found in *mop1-1* mutant in response to ABA when compared to *Mop1* wildtype (Table S1).

**Table 1:** Summary of RNA-seq libraries per genotype and treatment

Genotype	Treatment	Replicate	Total raw reads	Clean reads	Mapped reads	% Mapped reads	Uniquely mapped reads	% Uniquely mapped reads
*Mop1* wildtype	control	1	22307497	22181351	19983179	90.09	17478905	78.80
	control	2	22220268	22103764	19606039	88.70	17123786	77.47
	control	3*^a^*	25460004	25345274	22656140	89.39	20020232	78.99
	ABA	1	22263417	22135607	19700690	89.00	17356529	78.41
	ABA	2	23131739	23008377	20776564	90.30	18613777	80.90
	ABA	3	21771139	21657574	19680237	90.87	17650923	81.50
*mop1-1* mutant	control	1	24469219	24316810	21753818	89.46	19001155	78.14
	control	2	24142476	23989219	22240405	92.71	20196523	84.19
	control	3	23023844	22878557	19890617	86.94	17579883	76.84
	ABA	1	21775951	21606778	19832862	91.79	18099998	83.77
	ABA	2	22373108	22282587	20183567	90.58	17919656	80.42
	ABA	3	21583557	21464462	19414606	90.45	17055662	79.46

a Removed from analysis.

**Figure 1:**
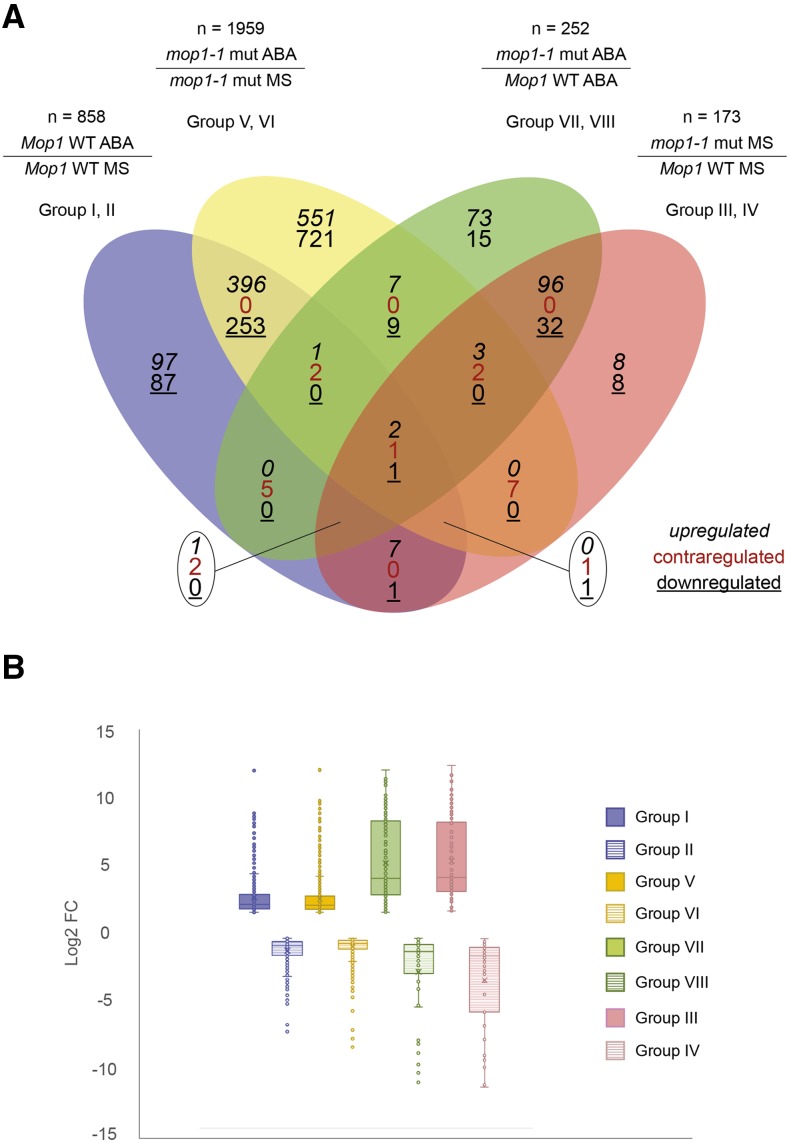
Genome-wide differential gene expression with log_2_ FC ≥ 0.95, FDR < 0.05. (a) Venn Diagram showing the overlap of differentially expressed genes (DEGs) specifying upregulated (black, italics), contra-regulated or genes that have diverse regulation polarity (red), and downregulated (black, underlined) genes in four pair-wise-comparisons. The Venn Diagram was generated using Vennplex (Cai *et al.* 2013). (b) Distribution of significant differential gene expression log2 fold change (FC) in four comparisons. Boxes encompass the upper and lower quartile log2 FC, the middle line depicts the median, the cross represents the mean, the whiskers show the maximum and minimum log2 FC values, and the dots outside the whiskers show the outliers.

Significant DEGs were identified in four pairwise comparisons as genes with a twofold expression change (log_2_ FC ≥ 0.95, FDR < 0.05) (Table 2). The DEGs in the four comparisons were further sub-divided into eight analysis groups based on gene expression patterns identified by making direct comparisons between genotypes and treatments (Table 2; File S1). For example, by comparing expression in wildtype plants in the presence and absence of ABA treatment, ABA-induced (Group I) and ABA-repressed (Group II) genes were detected. The total number of DEGs identified in all eight groups was 3,242 genes; however, 1,681 genes were found to be common to more than one analysis group, resulting in 1,561 DEGs unique to an individual analysis group (Table 2*)*. Analysis of the individual groups of unique DEGs revealed relationships between ABA-responsiveness and MOP1-mediated effects on transcriptional regulation in maize (Figure 1).

**Table 2:** Differentially expressed gene categories and analysis groups

Pair-wise comparison	Analysis group	Expression pattern	Significant *^a^* DEGs	2FC Significant *^a^* DEGs	Total Significant *^a^* DEGs	Total 2FC Significant *^a^* DEGs	Interpretation
*Mop1* wildtype ABA / *Mop1* wildtype MS	I	upregulated	606	506	1028	858	ABA response in wildtype
	II	downregulated	422	352			
*mop1-1* mutant MS / *Mop1* wildtype MS	III	upregulated	126	126	173	173	Response to loss of RdDM
	IV	downregulated	47	47			
*mop1-1* mutant ABA / *mop1-1* mutant MS	V	upregulated	1511	965	3118	1959	ABA response in RdDM deficient mutant
	VI	downregulated	1607	994			
*mop1-1* mutant ABA / *Mop1* wildtype ABA	VII	upregulated	197	194	256	252	Differential ABA-response in RdDM deficient mutant compared to wildtype
	VIII	downregulated	59	58			
Total DEGs					4575	3242	
Number of DEGs in more than one analysis group					2091	1681	
Number of DEGs in only one analysis group					2484	1561	

a Significant genes are DEGs with a p-value and FDR < 0.05.

In summary, several well-documented, abiotic-stress-associated responses were found to be ABA responsive in wildtype and *mop1-1* plants (Groups I and V; File S1). In addition to *Rab17*, notable examples include twelve of the twenty maize *Protein Phosphatase 2C-like class*, *group A (PP2C-A)* genes (Table S2) that are known to act as negative regulators of ABA signaling in Arabidopsis and other plant species (reviewed by Mehrotra *et al.* 2014).

Many of the DEGs identified in untreated *mop1-1* mutants relative to untreated *Mop1* wildtype (Groups III and IV) are comparable to published *mop1-1* expression datasets (Jia *et al.* 2009; Madzima *et al.* 2014) in spite of tissue-specific expression differences that likely exist between these studies. The maize *Dng103* (Zm00001d038302) gene, an ortholog of the Arabidopsis DNA glycosylase gene *Repressor of silencing1* (*Ros1*) (Morales-Ruiz *et al.* 2006), has been shown to be downregulated in *mop1-1* shoot apical meristems (SAMs) (Jia *et al.* 2009), immature ears (Madzima *et al.* 2014), and leaves (Williams *et al.* 2015). Based on our expression criteria for DEGs, *Dng103* did not appear in Group IV as was expected, however, downregulation of *Dng103* in *mop1-1* seedlings was confirmed by RT-qPCR (Figure S3). Interestingly, *Dng103* expression was highly variable across biological replicates, and the gene appears to be upregulated in response to exogenous ABA application in wildtype but not *mop1-1* plants (Figure S3).

Genes identified in Groups I and II (ABA-responsive in WT) and Groups III and IV (MOP1-dependent) are similar to previously reported transcriptomic studies of ABA in Arabidopsis (Seki *et al.* 2002; Matsui *et al.* 2008; Liu *et al.* 2018) and MOP1 in maize (Jia *et al.* 2009; Madzima *et al.* 2014)]. Therefore, we focus herein on the novel findings of their combined regulation, identified predominantly through analysis of Groups V-VIII DEGs (Table 2).

### Loss of MOP1 amplifies ABA transcriptional responses in maize seedlings

The largest numbers of DEGs from all eight analysis groups were observed in Groups V and VI (representing 60% of all DEGs), which were identified by comparing expression in *mop1-1* mutants exposed to exogenous ABA relative to untreated mutant siblings. Groups V and VI include 965 and 994 up- and downregulated genes, respectively (Table 2). Of the 1959 DEGs, one-third of these (658 genes) were also differentially expressed in wildtype plants in response to exogenous ABA (Groups I and II) (Figure 1A), suggesting that these are ABA-dependent genes. When comparing the magnitude of effect of ABA exposure on gene expression, both genotypes exhibit a similar average and distribution of fold-change in response to ABA treatment (Groups I, II, V, and VI; Figure 1B). However, a much larger number of genes meet the significance and fold-change thresholds established for these analysis groups in *mop1-1* homozygous plants compared to wildtype. This results in ∼2x more upregulated genes and ∼3x more downregulated genes in *mop1-1* mutant plants when compared to *Mop1* wildtype plants (Table 2), suggesting that the loss of MOP1 amplifies genome-wide ABA-induced changes in gene expression in maize.

### In the absence of MOP1, ABA induces differential expression of a unique subset of genes and distinct functional categories

A direct comparison of the ABA response in homozygous *mop1-1* plants *vs.* wildtype allows for the identification of 252 genes that are differentially expressed between these two genotypes in the presence of exogenous ABA (Groups VII and VIII; Table 2). Specifically, there were 194 up- and 58 downregulated DEGs in ABA-treated *mop1-1* compared to wildtype. Of these 252 genes, 128 were also included in Groups III and IV (Figure 1A), indicating that these genes were differentially expressed in *mop1-1* homozygous plants in a similar manner with or without ABA treatment.

Of the 252 genes, one third (88 genes) were uniquely differentially expressed in these groups and not detected by other comparisons. These 88 genes would not have been identified as differentially expressed if gene expression had not been analyzed in the absence of MOP1 and the presence of ABA simultaneously. Genes in this category include specific members of the abiotic stress-responsive LATE EMBRYOGENESIS ABUNDANT (LEA), dehydrins, Glucosyltransferase/Rab-like GTPase activator/Myotubularin (GRAM) and VQ gene families that were uniquely upregulated in response to ABA in *mop1-1* mutants (Table 3). The unique expression patterns of genes with predicted physiological and developmental roles suggests that plants experiencing a loss of MOP1 together with the induction of ABA responses may have biological consequences. To explore this further, the identity and predicted function of DEGs across the different analysis groups were characterized.

**Table 3:** Abiotic stress-responsive LEA, Dehydrins, GRAM and VQ genes uniquely upregulated in response to ABA in *mop1-1* mutant

*mop1-1* Mutant Unique			
Gene Family	Gene name	Gene ID	log_2_FC
Late Embryogenesis Abundant (LEA)	Embryonic protein DC-8	Zm00001d002360	3.83
	LEA5-D-like	Zm00001d040659	1.82
	putative desiccation-related protein *LEA14*	Zm00001d009700	1.04
	**-**	Zm00001d032620	1.04
Dehydrins	*Dhn3*	Zm00001d051420	1.56
	*COR410*	Zm00001d017547	2.01
GRAM	**-**	Zm00001d023664	1.56
	**-**	Zm00001d032636	1.15
VQ	*ZmVQ13*	Zm00001d00208	1.39
	*ZmVQ29*	Zm00001d015397	1.20
	*ZmVQ53*	Zm00001d046961	1.58
	*ZmVQ58*	Zm00001d023269	2.18

Gene ontology (GO) analysis was used to predict the biological processes (BP) of annotated genes in each of the 8 analysis groups. Only genes from Groups I, II, V, and VI met the enrichment criteria for GO analysis (see Methods). More than 85% of the genes were annotated in these groups, resulting in 17 GO terms for Group I, 11 for Group II, 22 for Group V and 28 for Group VI (Table S4).

**Figure 2:**
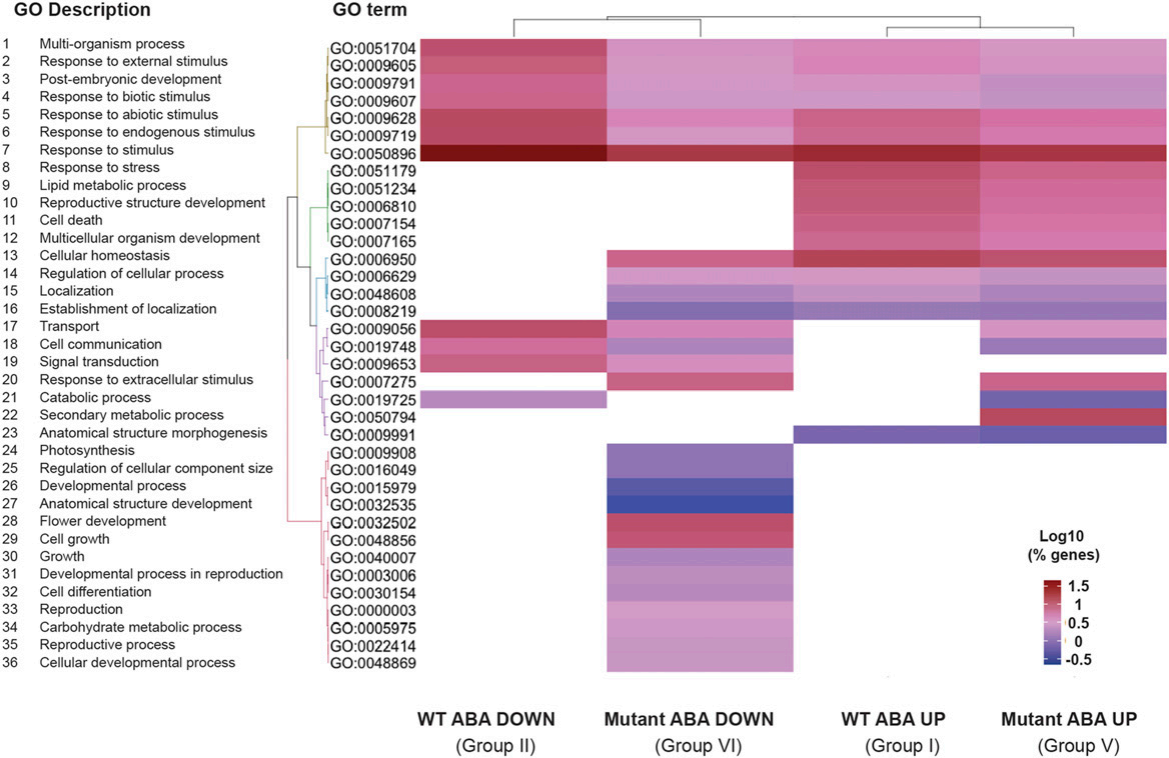
Enriched biological process Gene Ontology (GO) terms in differentially expressed genes (DEGs) (File S2). Comparisons were made between analysis Groups I, II, V, and VI (Table 2). Hierarchical clustering of log_10_ (% genes) of significant GO terms enriched in each expression comparison (FDR < 0.05, minimum of 10 genes per GO term). No color (white) shows no particular GO term enrichment in the dataset.

Consistent with response to ABA induction, the most prevalent GO term present across all four groups was ‘response to stimulus’ (GO:0050896; FDR ≤ 7E-05; File S2). Noteably, Group VI genes, which are downregulated in the *mop1-1* homozygous seedlings treated with ABA, were enriched with unique diverse GO terms such as growth, development, and reproduction, when compared to the other three groups (FDR ≤ 0.049; Figure 2). The enrichment of development-related genes that are differentially expressed between mutants and wildtype may be related to the pleiotropic developmental defective phenotypes observed in *mop1-1* and the many genes mis-regulated as a consequence of the loss of MOP1 activity (Dorweiler *et al.* 2000; Jia *et al.* 2009; Madzima *et al.* 2014).

### Hierarchically organized transcription factors are uniquely differentially expressed in response to ABA in the mop1-1 genotype

The amplified response to ABA observed in the *mop1-1* homozygous plants might be due to the combined consequences of direct and indirect effects. MOP1 and ABA treatment might each directly induce a subset of transcriptional changes that in turn activate a cascade of additional responses in maize. Transcription factors (TFs) are key regulators of the transcriptional activity of genes involved in networks that are fundamental for plant growth and development, as well as responses to stress stimuli, and might therefore induce secondary effects in such a cascade.

In response to ABA, a total of 90 transcription factors were induced in both the *Mop1* and *mop1-1* genotypes, or unique to one genotype (Table 4), including genes corresponding to the Zinc-finger (ZF), APETALA2/Ethylene-Responsive Element Binding Protein (AP2/EREBP), Basic Leucine Zipper (bZIP), Myeloblastosis (MYB), Homeobox (HB), and Heat Shock Factor (HSFTF) families of TFs (Table 4). For example, the AP2/EREBP superfamily is one of the largest known to play a critical role in abiotic stress responses in plants (Mizoi *et al.* 2012). Of the AP2/EREBP TFs identified in this study, most are upregulated in response to ABA (Table 4). Of these 90 TFs, 32 were found to be upregulated in response to ABA in both mutant and wildtype genotypes, whereas 51 TFs were upregulated in response to ABA only in *mop1-1* (Table 4). Other members of the same families of TFs were identified as downregulated in response to ABA treatment (72 TFs, Table 5). The majority of these (50 genes) were uniquely downregulated in the *mop1-1* mutant (Table 5), presumably through negative regulation directed by ABA-responsive and MOP1-silenced TFs. Another noteable observation from this analysis was that members of the NAC family of TFs, known to be responsive to ABA and dehydration (Nakashima *et al.* 2009), were exclusively differentially expressed in response to ABA in the *mop1-1* mutant (Tables 4 and 5).

**Table 4:** Transcription factor (TF) families upregulated in *Mop1* wildtype and *mop1-1* mutant in response to ABA (Groups I, V)

	*Mop1* Wildtype Unique		Common in WT and Mutant		*mop1-1* Mutant Unique	
TF Family	Gene name	Gene id	Gene name	Gene id	Gene name	Gene id
Ethylene-Responsive Element Binding (EREB): AP2/ERF, DREB/CBF, and RAV subfamily			*Ereb60*	Zm00001d032144	*Ereb13*	Zm00001d052152
			*Ereb106*	Zm00001d048991	*Ereb34*	Zm00001d039077
			*Ereb111*	Zm00001d053195	*Ereb69*	Zm00001d037941
			*Ereb122*	Zm00001d045204	*Ereb126*	Zm00001d043782
			*Ereb147*	Zm00001d043205	*Ereb133*	Zm00001d038320
			*Ereb156*	Zm00001d026447	*Ereb162*	Zm00001d038907
			*Ereb186*	Zm00001d008872	*Ereb179*	Zm00001d027924
			*Dhn1*	Zm00001d037894	*Ereb202*	Zm00001d005798
					*Dbf1*	Zm00001d032295
					*Abi13*	Zm00001d011639
					*Abi35*	Zm00001d017112
No apical meristem, ATAF1 and Cup-shaped cotyledon (NAC)					*Nactf25*	Zm00001d023294
					*Nactf30*	Zm00001d016950
					*Nactf40*	Zm00001d050893
					*Nactf60*	Zm00001d013003
					*Nactf118*	Zm00001d008399
Myeloblastosis (MYB)	*Glk16*	Zm00001d001936	*Myb26*	Zm00001d017383	*Myb22*	Zm00001d008528
	*Myb153*	Zm00001d019712	*Myb112*	Zm00001d046632	*Myb50*	Zm00001d015614
			*Myb159*	Zm00001d020408	*Myb1066*	Zm00001d037334
			*Myb162*	Zm00001d020457	*Myb132*	Zm00001d025864
					*Myb158*	Zm00001d036768
					*Myb166*	Zm00001d051267
					*Mybr24*	Zm00001d008808
					*Mybr55*	Zm00001d012285
					*Mybr58*	Zm00001d051480
					*Glk25*	Zm00001d010634
					*Fdl1*	Zm00001d022227
basic Leucine Zipper Domain (bZIP)	*Bzip45*	Zm00001d030577	*Bzip1*	Zm00001d043992	*Bzip9*	Zm00001d020025
			*Bzip4*	Zm00001d018178	*Bzip10*	Zm00001d023507
			*Bzip49*	Zm00001d031790	*Bzip27*	Zm00001d002143
			*Bzip95*	Zm00001d042721	*Bzip29*	Zm00001d034571
			*Bzip102*	Zm00001d043117	*Bzip76*	Zm00001d036736
			*Gbf1*	Zm00001d039065	*Bzip107*	Zm00001d024160
				Zm00001d042899		
WRKY	*Wrky38*	Zm00001d005622			*Wrky32*	Zm00001d028962
					*Wrky80*	Zm00001d008793
					*Wrky81*	Zm00001d043060
basic Helix-Loop-Helix (BHLH)	*Bhlh41*	Zm00001d007311	*Bhlh144*	Zm00001d004095	*Bhlh20*	Zm00001d005841
			*Bhlh160*	Zm00001d027987	*Bhlh132*	Zm00001d042482
			*Bhlh165*	Zm00001d049870	*Bhlh152*	Zm00001d016873
						Zm00001d048901
Homeobox (HB)			*Hb41*	Zm00001d017422	*Hb5*	Zm00001d027991
			*Hb52*	Zm00001d008869		Zm00001d002782
			*Hb62*	Zm00001d047995		
			*Hb70*	Zm00001d025964		
			*Hb78*	Zm00001d029934		
Heat Shock Factor TF (HSFTF)			*Hsftf18*	Zm00001d016255	*Hsftf3*	Zm00001d044259
			*Hsftf28*	Zm00001d046299		
Squamosa-promoter Binding Protein (SBP)			*Sbp29*	Zm00001d021573	*Sbp7*	Zm00001d052905
					*Sbp18*	Zm00001d012015
Nuclear Factor Y (NF-Y)			*Cadtfr6*	Zm00001d006813	*Ca2p1*	Zm00001d013501
Ethylene-Insensitive-Like (EIL)					*Eil8*	Zm00001d016924
Auxin/IAA			*Iaa44*	Zm00001d026480		
Other TFs	*Tcptf24*	Zm00001d008919			*Arftf29*	Zm00001d026540
		Zm00001d036533			*Bzr9*	Zm00001d027587
					*Mads45*	Zm00001d035053
					*Platz11*	Zm00001d017682

**Table 5:** Transcription factor (TF) families downregulated in *Mop1* wildtype and *mop1-1* mutant (Groups II, VI)

TF Family	*Mop1* WT Unique		Common in WT and Mutant		*mop1-1* Mutant Unique	
	Gene name	Gene id	Gene name	Gene id	Gene name	Gene id
EREB			*Ereb190*	Zm00001d026486	*Ereb46*	Zm00001d015759
					*Ereb143*	Zm00001d020540
NAC					*Nactf62*	Zm00001d034984
					*Nactf92*	Zm00001d050039
					*Nactf103*	Zm00001d002934
					*Nactf121*	Zm00001d021424
MYB	*Myb154*	Zm00001d047671	*Glk52*	Zm00001d026542	*Myb11*	Zm00001d022628
			*Myb42*	Zm00001d053220	*Myb23*	Zm00001d022259
			*Myb63*	Zm00001d052804	*Myb27*	Zm00001d044538
					*Myb38*	Zm00001d032024
					*Mybr78*	Zm00001d017782
					*Myb109*	Zm00001d023932
					*Myb131*	Zm00001d039496
					*Myb164*	Zm00001d031270
bZIP			*Bzip20*	Zm00001d012719	*Bzip53*	Zm00001d043420
			*Bzip84*	Zm00001d053988	*Bzip61*	Zm00001d015743
			*Bzip89*	Zm00001d016154		
WRKY					*Wrky20*	Zm00001d009698
					*Wrky100*	Zm00001d038761
BHLH			*Bhlh47*	Zm00001d034298	*Bhlh25*	Zm00001d044242
			*Bhlh173*	Zm00001d031665	*Bhlh30*	Zm00001d018056
					*Bhlh33*	Zm00001d005939
					*Bhlh97*	Zm00001d048309
					*Bhlh143*	Zm00001d047878
HB					*Glk53*	Zm00001d015407
					*Hb21*	Zm00001d046223
					*Hb122*	Zm00001d026537
						Zm00001d049000
HSFTF			*Hsftf25*	Zm00001d011406	*Hsftf13*	Zm00001d027757
NF-Y					*Ca5p15*	Zm00001d039581
EIL					*Eil2*	Zm00001d007188
IAA			*Iaa3*	Zm00001d033319		
Other TF			*Crr2*	Zm00001d026594	*Gbptf21*	Zm00001d040254
					*Irl1*	Zm00001d040173
					*Ptac12*	Zm00001d043325
					*Rack1*	Zm00001d038923
Zinc-Fingers		Zm00001d035195	*Dbb2*	Zm00001d002806	*C3h3*	Zm00001d039101
		Zm00001d037023		Zm00001d004439	*C3h21*	Zm00001d044074
				Zm00001d049625	*Col4*	Zm00001d045661
				Zm00001d048214	*Col10*	Zm00001d037327
				Zm00001d049525	*Id1*	Zm00001d032922
				Zm00001d016721	*Gata13*	Zm00001d012757
				Zm00001d006879		Zm00001d052883
						Zm00001d052918
						Zm00001d017871
						Zm00001d027684
						Zm00001d037974
						Zm00001d039579
						Zm00001d043422
						Zm00001d043728
						Zm00001d045454
						Zm00001d046289

A recent analysis of transcriptional responses to ABA and TF binding events in Arabidopsis suggests a hierarchical model of regulation, with three tiers of TFs and a set of target genes thought to directly impact physiological responses (Song *et al.* 2016). An implication of this model would be that a higher tier TF might induce a substantial cascade of effects by modulating expression of many genes in downstream tiers.

**Figure 3:**
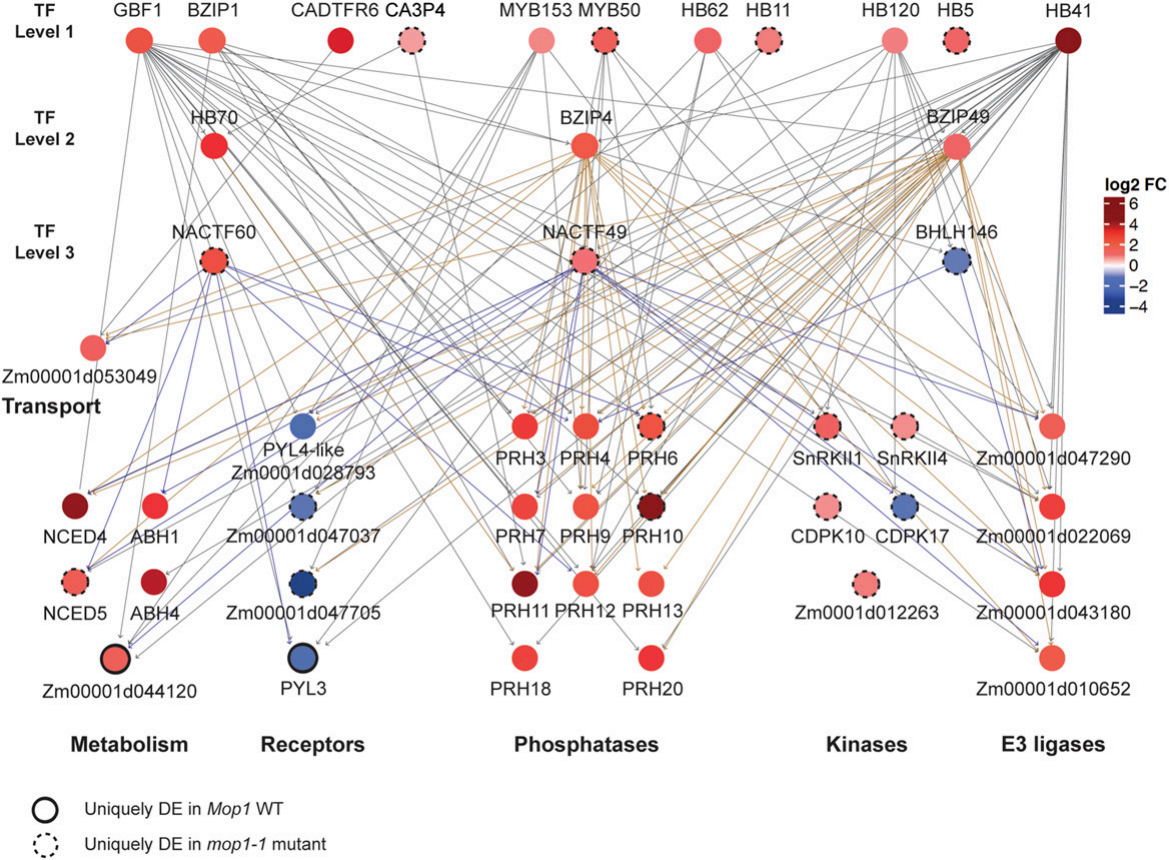
Model of maize transcription factor hierarchical network. Maize genes orthologous to Arabidopsis genes in a hierarchical, ABA-responsive network (Song *et al.* 2016) were identified among ABA-responsive DEGs in *Mop1* wildtype and *mop1-1* plants. Transcription Factors are separated in three levels based on the Arabidopsis hierarchical regulatory model (TF level 1, 2, and 3). Target genes are clustered by function (Transport, Metabolism, Receptors, Phosphatases, Kinases, and E3 ligases). Node color corresponds to log_2_FC. Nodes that lack a border are differentially expressed in both wildtype and mutant in response to ABA; nodes with a dotted border are differentially expressed only in the *mop1-1* mutant; and nodes with solid borders are differentially expressed only in wildtype.

To see if this hierarchical model could be adapted to MOP1- and ABA-mediated transcriptional responses, maize genes homologous to the TFs in the Song *et al.* (2016) model were identified in *Mop1* and *mop1-1* up- and downregulated genes using a list of maize orthologs of Arabidopsis genes generated in an earlier study (Huang *et al.* 2017) (File S3). Of the ten Tier 1 TF families in the Arabidopsis study, six were represented in our maize seedling dataset with one or more homologous genes (| log_2_ FC | ≥ 0.8, FDR < 0.05; File S3). Three of the six Tier 2 TFs and two of the five Tier 3 TFs had one or more homologs that were differentially expressed in maize seedlings exposed to ABA (| log_2_ FC | ≥ 0.91, FDR < 0.05; File S3). A total of 30 maize genes were identified as differentially expressed in maize seedlings and shared homology with the target genes identified by the hierarchical model (| log_2_ FC | ≥ 0.75, FDR < 0.05; File S3). A previously constructed gene regulatory network (GRN) (Huang *et al.* 2018) was used to predict the regulatory relationships between these maize genes in seed, shoot apical meristem, root, and leaf tissues (File S4). The connectivity of this network supports the idea that these genes are part of a regulatory network (Figure 3), and the gene expression responses observed in this study may result from several scenarios ranging from primary and secondary transcriptional responses to disruption or stimulus in a hierarchical network.

### Mop1 wildtype plants exhibit context-dependent ABA-induced cytosine methylation at specific loci, including particular families of transposons

The canonical model for MOP1-activity is based on a predicted role for MOP1 in RdDM-mediated gene silencing in which *mop1-1* homozygous plants display a (1) reduction in 24-nt siRNAs (Nobuta *et al.* 2008; Gent *et al.* 2014) and (2) reduced CHH methylation (Gent *et al.* 2014; Li *et al.* 2014).

To profile MOP1-dependent 24-nt siRNAs, we used publicly available datasets (Wang *et al.* 2017) to determine if upregulated genes were more likely to have hallmarks of direct regulation by MOP1 than downregulated genes. Genes that are differentially expressed in response to ABA in wildtype but not *mop1-1* homozygous plants might be examples of regulatory events that are ABA-responsive and MOP1-dependent. Two hundred (200) genes are uniquely differentially expressed in wildtype in response to ABA. These 200 genes include 105 upregulated and 95 downregulated ABA-responsive genes in *Mop1* wildtype that are not detected as differentially expressed in ABA-induced *mop1-1* mutants; identified from a comparison of Groups I and II *vs.* Groups V and VI (Figure 1A). Eighty-six percent (86%; 171 genes) of the MOP1-dependent ABA-responsive genes had a known gene identifier. Of these 171 genes, 26.08% (24/92 upregulated genes, Group I) and 26.58% (21/79 downregulated genes, Group II) showed a depletion of MOP1-dependent 24-nt siRNAs in their promoter regions in the *mop1-1* mutant when compared to *Mop1* wildtype, respectively (Table 6). Although potentially limited by tissue-specific differences between the siRNA datasets and expression analysis in this study, there was no apparent relationship between MOP1-dependent siRNAs and upregulation in *mop1-1* mutants.

**Table 6:** 24-nt siRNA depletion percentage in *mop1-1* mutant in the 5′ proximal promoter region (2 kb upstream of TSS) of differentially expressed genes

Pair-wise comparison	Group	Subgroup	No. of total genes with gene model in group/subgroup	No. of genes with siRNA in 5′ proximal promoter	% siRNA depleted genes in *mop1-1* mutant
*Mop1* wildtype ABA / wildtype MS	I	All genes	470	108	22.98
		Unique to WT	89	24	26.97
	II	All genes	304	70	23.03
		Unique to WT	78	21	26.92
*mop1-1* mutant MS /*Mop1* wildtype MS	III	All genes	57	17	29.82
	IV	All genes	32	12	37.50
*mop1-1* mutant ABA / mutant MS	V	All genes	894	213	23.83
		Unique to *mop1-1*	516	129	25.00
	VI	All genes	893	185	20.71
		Unique to *mop1-1*	668	136	20.36
*mop1-1* mutant ABA /*Mop1* wildtype ABA	VII	All genes	83	19	22.89
	VIII	All genes	44	15	34.09
Upregulated genes in *mop1-1* mutant	III, V, VII	All genes	977	231	23.64
		Unique to *mop1-1*	596	147	24.67
Downregulated genes in *mop1-1* mutant	IV, VI, VIII	All genes	938	200	21.32
		Unique to *mop1-1*	712	151	21.21
	**Genome-wide**	**All genes**	**44,301**	**7,844**	**17.71**

To profile cytosine methylation changes, *Mop1* wildtype and *mop1-1* mutant seedlings subjected to ABA treatment or MS control were also used in a sequence-capture bisulfite sequencing approach (Li *et al.* 2014). This experiment allowed us to survey ABA-induced cytosine methylation in maize, as well as to explore the role of MOP1-mediated RdDM in ABA-induced epigenetic responses. The captured regions were selected based on several criteria as previously described (Han *et al.* 2018) and included a total of 21,822 loci with a mean length of ∼285 bp, representing ∼15 Mb (<1% of the B73 maize genome), and were aligned to the B73v4 reference genome (Jiao *et al.* 2017).

**Figure 4:**
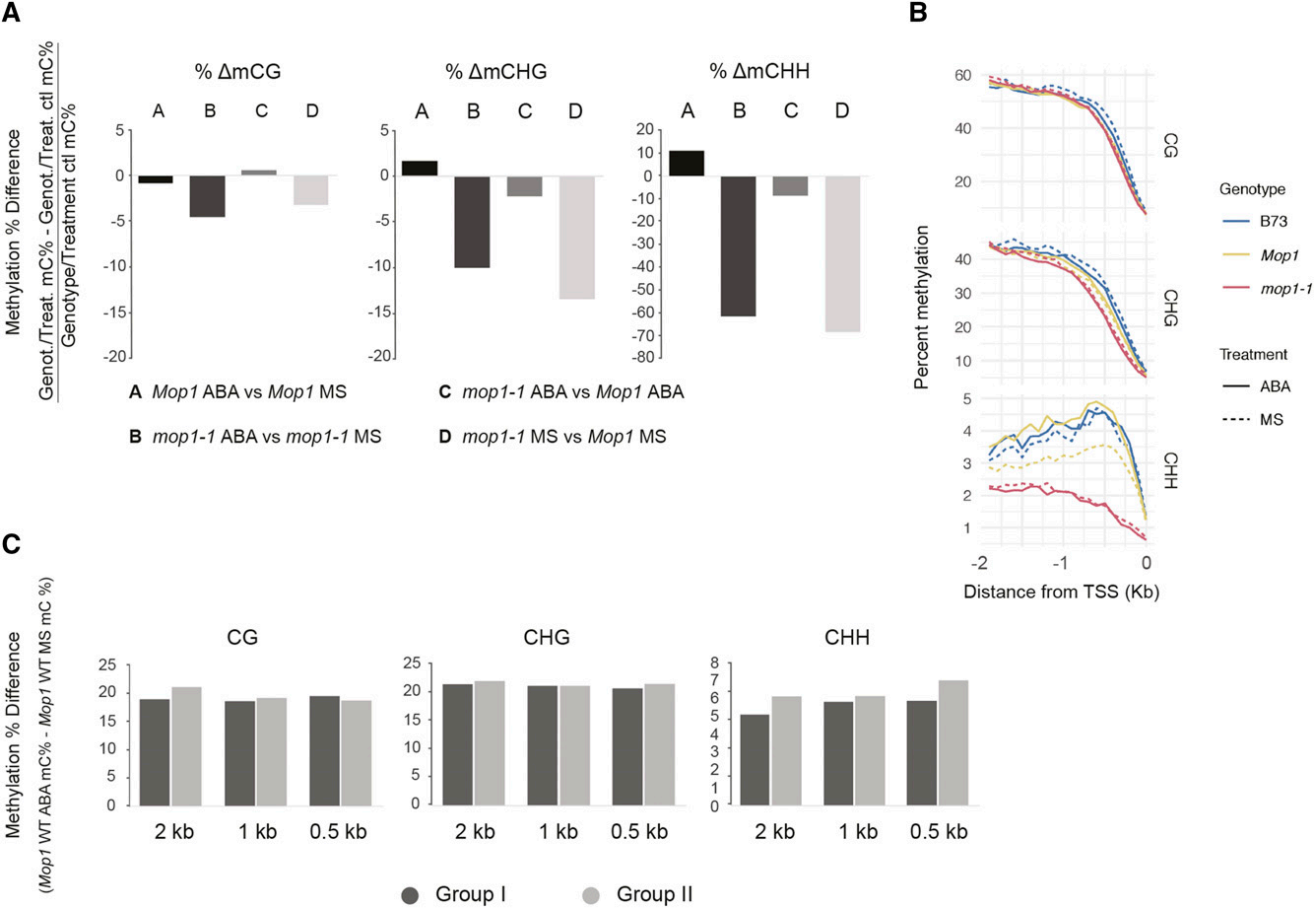
DNA methylation in *Mop1* wildtype and *mop1-1* mutant seedlings in response to abscisic acid. (A) Genome-wide methylation % difference in all sequence contexts (CG, CHG, and CHH) was calculated using the following equation ((Genotype/Treatment mC% - Genotype/Treatment control mC%) / Genotype/Treatment control mC%) for all regions in the genome. (B) Cytosine methylation % of the 2 Kb upstream region from the transcription start site (TSS) for three genotypes, B73 (blue), *Mop1* wildtype (yellow), *mop1-1* mutant (red) for two treatments, ABA (continuous) and MS (dotted). The mean methylation percentage (%) was calculated per genotype/treatment combination and context for all genes. (C) Methylation % difference in CG, CHG, and CHH context for *Mop1* wildtype ABA-upregulated (Group I) and ABA-downregulated (Group II) genes in three different promoter regions (2 Kb from TSS, 1 Kb from TSS, and a 0.5 Kb region including -0.25 to -0.75 Kb). Methylation % difference was calculated using the following equation (*Mop1* wildtype ABA mC% - *Mop1* wildtype MS mC%).

Consistent with the previously described loss of RdDM activity in *mop1-1* mutants (Gent *et al.* 2014; Li *et al.* 2014), the biggest difference in cytosine methylation in our control samples was observed in the CHH context (68% CHH reduction in *mop1-1* mutants relative to wildtype; compared to 14% CHG and 3% CG) (comparison D, Figure 4A). Interestingly, a slight increase in methylation in response to ABA treatment was observed in the CHG and CHH sequence contexts (1.6% and 11.1% respectively; comparison A, Figure 4A) in a unique manner in *Mop1* wildtype plants, suggesting ABA-induced cytosine methylation at specific loci. It is worth noting that *Mop1* wildtype plants introgressed into the B73 genome had lower than average CHH methylation compared to B73 plants treated with MS or ABA (Figure 4B) and the methylated CHH (mCHH) increase in the *Mop1* WT promoter region in response to ABA is similar to the untreated B73 methylation level. An ABA-dependent increase in CHH methylation was not observed for B73 plants in this experiment. Therefore, further experiments would be needed to separate the effects of pedigree-related epigenetic differences that may stem from a genomic exposure to *mop1-1* homozygous conditions and may take multiple generations to recover from.

A promoter analysis of genotype/treatment combinations in all three cytosine methylation sequence contexts shows an increase in DNA methylation in the CHH context in ABA-treated *Mop1* wildtype when compared to MS-treated wildtype plants (Figure 4B). An additional promoter analysis correlating the methylation percentage in this region for ABA-responsive DEGs and non-DEGs was performed (Figure S4). We observed that wildtype plants had higher CHH methylation within the first 1000 bp upstream from the TSS of ABA-responsive DEGs (Groups I, II, V and VI). This ABA-induced DNA methylation change is not observed as a response to this hormone in the *mop1-1* mutant, which suggests that MOP1 might be necessary for an epigenetic environmental response in maize seedlings (Figure 4B, Figure S4). The percent differences in DNA methylation for comparison A were measured in the promoter region of wildtype ABA-responsive DEGs (Groups I and II). Regions of 2 kb (0 to -2.0 Kb), 1 kb (0 to -1.0 Kb), and 500 bp (-0.25 to -0.75 Kb) upstream of TSS were assayed (Figure 4C, File S9). The average methylation differences between ABA-up (Group I) and downregulated (Group II) genes becomes more apparent in the smaller promoter region (500 bp), closer to the TSS in the CHH context, suggesting a dynamic transient methylation change as a response to ABA levels that are correlated with gene expression regulation (Figure 4C). A GO term analysis of Group II genes that show an increase in CHH methylation in their promoter region identified four biological process GO terms related to response to endogenous stimulus (GO:0009719, FDR 6.1 e-5; GO:0009725, FDR 6.1 e-5; GO:0010033; FDR 0.0007, GO:0042221, FDR 0.02) and four molecular function GO terms related to hydrolase and transferase activity of glycosyl compounds (GO:0004553, FDR 0.0011; GO:0016798, FDR 0.0015; GO:0016758, FDR 0.016; GO:0016757, FDR 0.024). The increase in CHH methylation of this group of genes during ABA treatment suggests the transcriptional epigenetic regulatory machinery in maize seedlings during environmental stimuli tends to direct gene silencing toward mechanisms involved in metabolism in order to efficiently activate processes of stress response and recovery.

**Figure 5:**
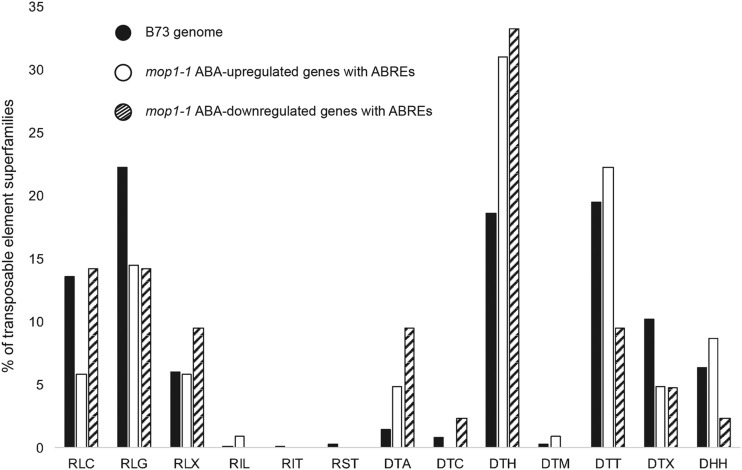
Percentage of transposable elements (TEs) within each TE superfamily. TEs detected within 2 kb upstream or downstream of genes with ABREs or CEs that were upregulated (white) or downregulated (stripes) in *mop1-1* mutant plants treated with ABA represented multiple TE categories. The TEs analyzed include Class I of LTR retrotransposons Copia (RLC), Gypsy (RLG), and unclassified (RLX), LINES L1 (RIL) and RTE (RIT), and SINES (RST) as well as class II of DNA TIR transposons hAT (DTA), CACTA (DTC), Pif/Harbinger (DTH), Mutator (DTM), Tc1/Mariner (DTT), and unclassified TIR (DTX), and the Helitron superfamily (DHH). The abundance of the same TE families across the B73 genome (black; 338,224 TEs) was used as a comparison against *mop1-1* ABREs/CEs-containing ABA-upregulated (103 TEs) and downregulated (42 TEs) genes. Bars depict the percentage (y-axis) of the TE categories (x-axis) in each subset that belong to each TE family.

MOP1 function has been previously correlated with genomic regions adjoining particular types of transposable elements and genic regions (Madzima *et al.* 2014; Li *et al.* 2015a), suggesting that this regulatory pathway functions to maintain distinct transcriptional activities within genomic spaces, and that loss of MOP1 may modify the responsiveness of some loci to other regulatory pathways. We analyzed the enrichment of individual TE families in regions adjacent to ABA-regulated genes in the *mop1-1* mutant that contain ABA-responsive *cis*-regulatory elements (ABREs) (177 and 67 DEGs from Groups V and VI, respectively). Approximately 50% of the TEs found within 2 kb upstream of the transcription start sites (TSS) or 2 kb downstream of the transcription termination sites (TTS) of genes predicted to be common MOP1-ABA regulatory gene targets belong to the Pif/Harbinger (DTH) and Tc1/Mariner (DTT) superfamilies of the class II DNA TIR transposons. This is consistent with previous reports (Gent *et al.* 2013) and shows a considerable change in the number of DTH and DTT transposons at these loci when compared to the B73 genome (Figure 5, File S10). In our study, the DTH superfamily of TEs was 12% and 15% more abundant near upregulated and downregulated genes, respectively, compared to the distribution of this TE category across the B73 genome. Additionally, the DTT elements were half as abundant near ABRE-containing ABA-downregulated genes in the *mop1-1* mutant than near ABA-upregulated genes or across the B73 genome. These findings suggest that loss of MOP1 may modify how responsive these genes are to other regulatory pathways like ABA, due to changes in TE transcriptional maintenance at these loci.

### Gene expression responses to ABA involve coordination of cis-elements, CHH methylation, and MOP1-mediated effects

Genes directly induced or repressed by ABA are expected to have canonical ABA *cis*-regulatory sequences that are recognized by ABA-phosphorylated TFs. To predict primary transcriptional targets of ABA, we searched for ABRE and CE *cis*-regulatory elements within the putative promoters of DEGs in ABA-treated wildtype (Groups I and II) and mutant (Groups V and VI) samples.

Out of the total 858 genes in Groups I and II, this analysis was limited to 774 genes with a named gene model in the annotated maize genome version 40 (Zea_mays.AGPv4.40.gtf, EnsemblPlants; (Kersey *et al.* 2018)) (File S5). One hunded thirteen (113) genes were identified as ABA primary targets: 94 were identified to have potential ABRE and CE sites within the 2 kb sequence upstream of the transcription start sites (TSS) using the MEME-suite (Bailey *et al.* 2015) (File S5) and the remaining 19 genes were identified through a literature search of genes empirically demonstrated to be directly regulated by ABA in previous published studies (File S5). Based on the GRN (Huang *et al.* 2018), 23 of these 113 genes are predicted to regulate the expression of a collective group of 501 genes. These 23 genes are TFs (14 genes) and Prh/PP2CA (8 genes), with one of unknown category. Within these 501 genes, 75 were also predicted to contain ABA-responsive *cis*-regulatory elements and be directly regulated by ABA, while 426 genes were exclusively predicted to be secondary targets (File S5).

We identified a negative correlation between transcription and CHH methylation specifically in downregulated ABRE/CE containing genes (ABA-primary targets) in ABA-treated *Mop1* wildtype seedlings. Of 113 ABA-primary targets, 92 were upregulated and 21 were downregulated in ABA-treated wildtype plants. Of the 21 downregulated ABA-primary targets, 14 exhibited an increased in CHH methylation, in contrast to the upregulated primary ABA-induced genes, where no change in CHH methylation was observed for any of the 92 genes. In *mop1-1* plants, 5 of these 14 genes are not downregulated nor do they exhibit CHH methylation in the ABRE proximal regions. This suggests that a subset of genes may be methylated and downregulated by a MOP1-dependent pathway in response to ABA. Induction of ABA-dependent MOP1-mediated methylation is also revealed by the increase in CHH methylation around TSS in response to ABA in *Mop1* wildtype, but not *mop1-1* mutant plants (Figure 4).

A total of 244 DEGs in ABA-treated *mop1-1* mutants were predicted to be ABA primary response genes based on the performed motif enrichment analysis and previously published experiments (File S5). Using these primary targets as predicted regulators in the GRN (Huang *et al.* 2018), differential expression of secondary targets by these primary targets could explain the change in expression of the 1,362 out of 1,542 genes that were not predicted to be primary response genes (File S5). Collectively, primary responses and their predicted secondary targets account for 90% of ABA-induced changes in gene expression in the *mop1-1* mutant, suggesting that a cascade of regulatory effects largely explains the combined effects of a loss of MOP1 and exposure to ABA.

## Discussion

In response to changing and potentially growth-limiting environmental conditions, plants have evolved adaptive physiological mechanisms that are often linked to activation or repression of gene expression. Extensive research in the field has uncovered an increasingly complex network of regulation that includes cross talk between many different types of stressors, phytohormones, and regulatory mechanisms (reviewed by kundu and Gantait 2017; Dangi *et al.* 2018). Important components of the regulation of gene expression in plants include epigenetic and ABA-induced transcriptional regulation (reviewed by Vishwakarma *et al.* 2017; Annacondia *et al.* 2018). Plant responses to stress are often non-additive, and simultaneous exposures to multiple stimuli have been documented to induce changes in gene expression that are distinct from either stimulus individually (Mittler 2006; Atkinson and Urwin 2012). Recent evidence suggests a particular importance of siRNA-mediated responses to stress-induced changes in gene expression (reviewed by Khraiwesh *et al.* 2012; Wang and Chekanova 2016). Therefore, this study sought to understand how *mop1-1* plants, known to have reduced 24-nt siRNA levels in maize (Nobuta *et al.* 2008), would respond to ABA at the level of gene expression. The patterns of gene expression observed in this study support a hierarchical model of gene expression similar to that proposed to explain environmental stress responses in the model plant Arabidopsis, which includes 3 interconnected tiers of transcriptional regulators that, directly and indirectly, regulate the expression of many ABA-responsive target genes (Song *et al.* 2016). The work reported herein also supports the integration of MOP1-mediated regulation into these hierarchical plant responses and coordinate epigenetic and stimulus-driven regulation.

Our analysis of gene expression in V3 maize seedlings in the presence and absence of exogenous ABA treatment uncovered genome-wide changes in gene expression. Many of these changes were genotype independent and included many known examples of ABA-responsive genes in plants. Consistent with known pleiotropic phenotypes in *mop1-1* homozygous plants, the analysis also revealed that *mop1-1* mutants did not respond to ABA treatment in an identical manner to their wildtype siblings. These differences could be explained by the disruption of a hierarchical regulatory pathway, with a cascade of effects on gene expression, MOP1-dependent responses to ABA in maize, and combined direct and indirect effects of MOP1 and ABA response acting in synergy to create non-additive gene expression profiles across the genome. The largest number of differentially expressed genes was detected in *mop1-1* plants that had been exposed to exogenous ABA. This suggests that some distinct genes become more responsive to ABA in the absence of MOP1, while some ABA-responsive genes are unresponsive to ABA in the absence of MOP1. These relationships may be evidence of coordination between ABA-mediated and MOP1-mediated regulatory pathways and reinforce the idea that epigenetic regulation is crucial to plant response and adaptation to abiotic stress. These results also support the idea of the presence of epigenetically silenced genes that are “hidden” from ABA-responsiveness in *Mop1* wildtype. This cryptic variation is epigenetically “uncovered” in the *mop1-1* mutant in response to ABA as differentially expressed epi-alelles.

To estimate direct and indirect MOP1-mediated effects, we explored the distribution of MOP1-dependent siRNAs in the predicted promoter regions of DEGs. The genes that are downregulated in *mop1-1* plants are often regarded as indirect targets of a loss of MOP1 because of the canonically accepted silencing activity of MOP1 dependent pathways. However, these genes also appear to be enriched for 5′ proximal MOP1-dependent siRNAs; one potential explanation for this would be the coordinated activity of RdDM and ROS1-dependent demethylation to maintain gene activity, as has been described in Arabidopsis (Lei *et al.* 2015; Williams *et al.* 2015). Some of the RdDM targets are promoter regions that can be actively demethylated by ROS1 (Agius, Kapoor, & Zhu 2006; Morales-Ruiz *Et Al*. 2006; Penterman, Zilberman, *Et Al*., 2007). RDR2 is an essential component of RdDM and is the MOP1 homolog in Arabidopsis. In the *rdr2* background, CG hypermethylation was found in the promoter region of downregulated ROS1 target genes, suggesting ROS1 demethylation is associated with RDR2 (Penterman *Et Al*. 2007). Many stress-responsive genes are also downregulated in a triple DNA demethylase mutant (Le *Et Al*. 2014). Consistently, ROS1 is required for transcriptional activation via demethylation of specific ABA-inducible genes (Kim *et al.* 2019). These genes contain TEs and show DNA methylation changes in their promoters, suggesting that RdDM might mediate regulation of stress-responsive genes in a manner that involves DNA demethylation (Le *Et Al*. 2014).

There are multiple genes in the maize genome with amino acid sequence similarity to Arabidopsis ROS1 (reviewed by LMadzima, Sloan *et al.* 2014), and at least one of them appears to be downregulated and not responsive to ABA induction of expression in *mop1-1* seedlings. It is therefore possible that some of the genes identified in this analysis are additional examples of coordinate regulation by RdDM-gene silencing and demethylation-associated transcriptional activation, and that disruption of this coordinated regulation relates to some of the phenotypes exhibited by *mop1-1* homozygous plants.

It is possible that the upregulated genes in *mop1-1* plants for which MOP1-dependent siRNAs were not identified are indirect targets of MOP1, or that siRNAs exist but were not detected in the datasets used for this analysis because of tissue-specific differences in siRNA populations or incompletely annotated gene models. Based upon the diverse and diffuse gene expression phenotypes observed, it is likely that there are different explanations for the responses of different groups of genes.

The sequence-capture methylation analysis allowed us to identify a handful of examples in which there is an overlap between ABA and RdDM regulatory mechanisms. Previous studies have reported an increase in non-CG methylation (CHG and CHH) in intergenic TE sequences in response to osmotic stress, which induces ABA biosynthesis and is associated with a transient epigenetic adaptation to environmental cues (Wibowo *et al.* 2018). This is consistent with the fact that CHH methylation is not stochastic and has been observed to change as a response to abiotic stress in plant species (Dubin *et al.* 2015; Secco *et al.* 2015; Wibowo *et al.* 2018). The role of ABA in DNA methylation/demethylation and gene expression processes in maize is not yet understood.

Interpreting the complexity of plant responses to the environment is essential to a complete understanding of plant growth, development and physiology, with particular regard to crop yield and agricultural challenges. By perturbing phytohormone levels and an essential component of epigenetic regulation, some unique plant responses were observed. These results shed some insight onto the nature of combinatorial, and potentially synergistic, regulation of gene expression in maize.
